# A person-centered perspective on the combined DSM-5 AMPD/ICD-11 personality model: Utility, relationship with the categorical personality disorder model, and capacity to differentiate between levels of identity functioning

**DOI:** 10.3389/fpsyt.2022.1006842

**Published:** 2022-10-17

**Authors:** Tim Bastiaens, Annabel Bogaerts, Koen Luyckx, Dirk Smits, Laurence Claes

**Affiliations:** ^1^University Psychiatric Center KU Leuven, Leuven, Belgium; ^2^Faculty of Psychology and Educational Sciences, KU Leuven, Leuven, Belgium; ^3^Department of Research and Project Management, University of the Free State, Bloemfontein, South Africa; ^4^UNIBS, Odisee University College, Brussels, Belgium; ^5^Faculty of Medicine and Health Sciences, University of Antwerp, Antwerp, Belgium

**Keywords:** PID5BF + M, personality disorder, person-centered, personality types, personality clusters

## Abstract

**Background:**

Both the ICD-11 classification of Personality Disorders and the DSM-5 Alternative Model for Personality Disorders (DSM-5 AMPD) conceptualize personality pathology in a dimensional way, but differ in the way they carve up their respective pathological personality domains. Recently, a combination of ICD-11 and DSM-5 AMPD descriptive pathological personality traits, the Modified Personality Inventory for DSM-5—Brief Form Plus (PID5BF + M), was developed.

**The current study:**

We investigated the utility of the additional ANANKASTIA domain (not represented in the DSM-5 AMPD) as well as of the additional PSYCHOTICISM domain (not represented in the ICD-11 model) in the identification of meaningful pathological personality domain clusters based on the PID5BF + M. Next to the classical 2- and 3-cluster solutions, we examined whether the presence of the additional ANANKASTIA domain would also gave rise to a meaningful 4-cluster solution. We then validated these clusters by investigating differences between them in mean DSM-5 Section II cluster A, B, and C personality disorder scores. Finally, we investigated whether cluster membership was able to differentiate between levels of identity functioning, a key feature of personality disorder severity in both the ICD-11 model and the DSM-5 AMPD.

**Materials and methods:**

We used a Flemish community sample of 242 participants, and applied k-means cluster analyses in a two-step manner on PID5BF + M domains to investigate 2-, 3-, and 4-cluster solutions. We used MANOVAs to examine differences between clusters in PID5BF + M domains, DSM-IV/DSM-5 Section II Assessment of Personality disorders (ADP-IV) cluster A, B, and C scores, and Self-Concept and Identity Measure (SCIM) scores.

**Results:**

Cluster analyses on PID5BF + M pathological personality domains (1) revealed meaningful 2-, 3-, and 4-cluster solutions, with the 4-cluster solution explaining the most variance in the clustering variables, (2) allowed to identify a classical Overcontrolled cluster which DSM-5 AMPD PID-5 does not, and (3) demonstrated the utility of representing ANANKASTIA and DISINHIBITON as separate pathological personality domains. PID5BF + M clusters (5) were informative of DSM-5 Section II cluster A, B, and C personality disorder scores and (6) showed different levels of clinical-developmental Identity functioning.

**Conclusion:**

Current results demonstrate the utility of a combined ICD-11/DSM-5 AMPD view from a person-centered perspective.

## Highlights

- Cluster analyses on combined ICD-11/DSM-5 AMPD pathological personality trait domains reveal meaningful 2-, 3-, and 4-cluster solutions, with the 4-cluster solution explaining the most variance in the clustering variables.
- In the 3- and the 4-cluster solution, the separate ANANKASTIA domain of the PID5BF + M allows to identify a classical Overcontrolled type which DSM-5 AMPD PID-5 does not.
- The 4-cluster solution demonstrates the utility of representing ANANKASTIA and DISINHIBITON as independent domains, as the fourth personality cluster simultaneously exhibited high DISINHIBITION and high ANANKASTIA.
- PID5BF + M clusters are informative of DSM-5 Section II Cluster A, B, and C personality disorder scores.
- PID5BF + M clusters show different levels of clinical-developmental Identity functioning, a core feature of DSM-5 AMPD and ICD-11 personality disorder severity.
- Current results demonstrate the utility of a combined ICD-11/DSM-5 AMPD view from a person-centered perspective.

## Introduction

### Three models for diagnosing personality disorders

Currently, three mainstream descriptive classification systems for personality disorders exist: the categorical Diagnostic Statistical Manual Section II model (DSM-5) (1), the DSM-5 Alternative Model for Personality Disorders (AMPD) (1), and the ICD-11 classification of Personality Disorders (ICD-11) (2). While the DSM-5 Section II model delineates the classical ten personality disorders (grouped into clusters A, B, and C) in a categorical way, both the DSM-5 AMPD and the ICD-11 model conceptualize personality pathology in a dimensional manner. Also, the latter two models both distinguish between personality disorder severity as the core feature of personality pathology, and a number of pathological personality domains that can be brought in alignment with the general personality domains of the classical Five Factor Model (FFM) (3; Table 1), considered the descriptive manifestation of personality pathology.

**TABLE 1:** FFM, DSM-5 AMPD, ICD-11 classification of personality disorders, and PID5BF + M (pathological) trait descriptors.

FFM	DSM-5 AMPD	ICD-11	PID5BF + M
Emotional stability (+)/Neuroticism (−)	Negative affectivity	Negative affectivity	Negative affectivity
Extraversion (+)	Detachment (−)	Detachment (−)	Detachment (−)
Agreeableness (+)	Antagonism (−)	Dissociality (−)	Dissociality (−)
Conscientiousness (+)	Disinhibition (−)	Disinhibition (−)	Disinhibition (−)
		Anankastia	Anankastia
Openness	Psychoticism	/	Psychoticism

First, with regard to personality disorder severity, both the ICD-11 and the DSM-5 AMPD define levels of Dysfunction (ICD-11) or levels of Impairment (DSM-5 AMPD) including problems in Self-functioning. In ICD-11, Self-functioning refers to the constructs Identity, Self-worth, Accuracy of Self-view, and Self-direction. In DSM-5 AMPD, Self-functioning refers to (the DSM-5 AMPD definition of) Identity, and to Self-direction.

Second, with regard to the descriptive manifestation of personality pathology, the DSM-5 AMPD and the ICD-11 model differ substantially in the way they carve up their respective pathological personality domains, as shown in Table 1. Whereas the DSM-5 AMPD includes a PSYCHOTICISM domain, the ICD-11 model does not, and whereas the ICD-11 model distinguishes between a DISINHIBITION domain (i.e., an inclination to behave rashly following immediate internal of external stimuli) (2) and an ANANKASTIA Domain (i.e., a propensity for perfection, moral standards, conformity, and control over behavior of self and others) (2), the DSM-5 AMPD subsumes both under one broad DISINHIBITION domain (including negatively keyed ANANKASTIA facets). Recent studies have documented the strengths and weaknesses of both architectures (4, 5), and have pointed to the problematic DSM-5 AMPD conceptualization of the DISINHIBITION domain on the one hand (6), and to the utility of a PSYCHOTICISM domain on the other (7). In reply to these and other concerns, Kerber et al. (8) have developed the Personality Inventory for DSM5—Brief form Plus (PID5BF+), a measure integrating DSM-5 AMPD and ICD-11 pathological personality trait descriptors. The PID5BF + was subsequently adapted further into the Modified Personality Inventory for DSM-5—Brief Form Plus (PID5BF + M) (4), and includes a revised ANANKASTIA domain separate from a DISINHIBITION domain, while also including a PSYCHOTICISM domain (Table 1). The PID5BF + M has been validated in 15 countries (4).

### Overlap between pathological personality domains and personality disorder severity scores

While both the DSM-5 AMPD and the ICD-11 models are considered a useful and necessary departure from previous personality disorder conceptualizations, they are not without room for improvement. At least at the measurement level, pathological personality domains and personality disorder severity scores have been found to exhibit important overlap. For example, Sleep et al. (9) found significant correlations between DSM-5 AMPD Identity and DSM-5 AMPD Self-direction on the one hand, and DSM-5 AMPD NEGATIVE AFFECTIVITY (*r* = 0.69 and *r* = 0.53), DSM-5 AMPD DETACHMENT (*r* = 0.51 and *r* = 0.55), DSM-5 AMPD DISINHIBITION (*r* = 0.53 and *r* = 0.61), and DSM-5 PSYCHOTICISM (*r* = 0.43 and *r* = 0.44) on the other. In contrast, DSM-5 AMPD ANTAGONISM correlated *r* = 0.27 and *r* = 0.29 with DSM-5 AMPD Identity and DSM-5 AMPD Self-direction, respectively (9). Recent research on the ICD-11 model has demonstrated parallel results. For example, Clark et al. (10) found significant associations between the ICD-11 Self-functioning and ICD-11 NEGATIVE AFFECTIVITY (*r* = 0.83), ICD-11 DETACHMENT (*r* = 0.45), and ICD-11 DISINHIBITION (*r* = 0.57), while ICD-11 DISSOCIALITY and ICD-11 ANANKASTIA correlated *r* = −0.01 and *r* = 0.25 with ICD-11 Self-functioning, respectively. As a consequence, at least with regard to the DSM-5 AMPD, some have suggested the exclusive use of the pathological personality trait descriptors (11, 12), whereas others have advocated a primary focus on personality disorder severity measures in future conceptualizations (13).

### A person-centered perspective

With the aforementioned studies pertaining to a variable-centered view, which focuses on each of the pathological personality domains separately, a person-centered view can help us gain more insight into the manifestation of specific configurations of pathological personality domains, and how these configurations relate to personality disorder severity. Classical findings of person-centered research using FFM personality domains have typically yielded 2- to 5-cluster solutions (14), with an overall-low and an overall-high cluster in 2-cluster solutions, and a Resilient cluster (showing moderate to low NEUROTICISM, moderate CONSCIENTIOUSNESS, and moderate to high AGREEABLENESS and EXTRAVERSION), an Overcontrolled cluster (showing high NEUROTICISM and high CONSCIENTIOUSNESS), and an Undercontrolled cluster (displaying high NEUROTICISM, low AGREEABLENESS, and low CONSCIENTIOUSNESS) in 3-cluster solutions (15, 16). These three clusters have been proven replicable across populations, and clinically useful in predicting long term personality functioning, mental health, and treatment success (14). Resilient persons have been documented as cooperative, socially skilled, and adaptive in the face of stressful situations (17). Overcontrolled persons have been typified as rigid with regard to control and at risk for internalizing problems (18). Undercontrolled persons typically are impulsive, aggressive, and show notable difficulties in emotion regulation (17). Of note, studies in different populations have generally shown a gradual increase in DSM-5 Section II personality disorder scores according to Resilient, Overcontrolled, and Undercontrolled cluster membership respectively. Also, Undercontrollers have been found to exhibit higher DSM-IV/DSM-5 Section II cluster A (odd-eccentric cluster) and cluster B (dramatic-emotional-erratic) personality disorder scores, and Overcontrollers higher Cluster C (anxious-avoidant-fearful cluster) scores (19–21).

More recently, Fisher and Robie (22), using Latent Profile Analysis, distinguished between an highly adaptive cluster (high EMOTIONAL STABILITY, high AGREEABLENESS, high EXTRAVERSION, and high CONSCIENTIOUSNESS), an adaptive cluster (with intermediate scores on all four personality dimensions), and a maladaptive cluster (displaying low EMOTIONAL STABILITY, low AGREEABLENESS, low EXTRAVERSION, and low CONSCIENTIOUSNESS). In parallel, using model-based cluster analysis on the Personality Inventory for DSM-5 (PID-5) (23), Bastiaens et al. (24) found evidence for a six-cluster solution, composed of a Very Resilient cluster (very low scores on all five PID-5 pathological personality domains), a Resilient cluster (low scores on all five PID-5 pathological personality domains), and an Undercontrolled cluster (overall high scores). In the absence of a CONSCIENTIOUSNESS/ANANKASTIA domain in the DSM-5 AMPD pathological personality trait descriptors (Table 1), the three former clusters were supplemented with an Anxious-Agreeable cluster (High NEGATIVE AFFECTIVITY, low ANTAGONISM), and an Anxious-Detached cluster (high NEGATIVE AFFECTIVITY, very high DETACHMENT). Lastly, a sixth, Confident-Disagreeable cluster was found, characterized by low NEGATIVE AFFECTIVITY, high DISINHIBITION, and very high ANTAGONISM. Although specifically confined to patients seeking bariatric surgery, Riegel et al. (25) very recently used the PID5BF+ (8) to delineate a 3-cluster solution using the DSM-5 AMPD pathological personality domains, and paralleling this to the 3-cluster solution emerging when using the ICD-11 pathological personality domains. In both views, the 3-cluster solution was composed of a low, a middle, and a high scoring cluster on all pathological personality domain scores. However, the authors did not report on the DSM-5 AMPD/ICD-11 combined view in the strict sense (i.e., cluster analysis results including the combined six domains). Second, the PID5BF+ does not fully capture the ICD-11 ANANKASTIA domain (8), which therefore has given rise to further modifications and resulted in the PID5BF + M (4).

### Personality domain clusters and personality disorder severity

Earlier studies (26, 27) have demonstrated how personality domain cluster membership is informative of personality disorder severity, with Undercontrolled clusters displaying the highest levels of severity, Resilient clusters the lowest, and Overcontrolled clusters situated in-between. Recently, in line with Fisher and Robie (22) findings using FFM personality domains, Bastiaens et al. (24) found that (1) the PID-5 Undercontrolled cluster showed significantly more problematic scores on all the Severity Indices of Personality Pathology (SIPP-118) (28) in comparison to the Very Resilient and the Resilient cluster, with (2) the Anxious-Agreeable cluster scoring in-between, and with (3) the Anxious-Detached cluster scoring within the range of the Undercontrolled cluster. Finally, in their treatment-seeking sample for bariatric surgery, Riegel et al. (25) also investigated the relationship between PID5BF + clusters and personality disorder severity, finding higher personality disorder severity scores in the cluster that exhibited the highest overall PID5BF + domain scores. However, as a stated limitation by the authors (25), they did not assess personality disorder severity with a separate measure.

### Identity as a shared, core feature of personality disorder severity in the DSM-5 AMPD and the ICD-11 model

Starting from the variable-centered findings by Sleep et al. (9) and Clark et al. (10), it is interesting to study the relationship between personality clusters and Identity as a shared, core feature of personality disorder severity in the DSM-5 AMPD and the ICD-11 model. The Self-Concept and Identity Measure (SCIM) (29, 30) takes a clinical-developmental approach to the construct of identity, including its adaptive next to its non-adaptive aspects. In addition, as Kaufman et al. (29) state, the SCIM conceptualization of identity allows for a view on the core sense of self (31), rather than an assessment of potential consequences of identity problems, like experiencing oneself as uncertain about one’s sexual orientation—which may in fact have other causes as well (29). The SCIM differentiates between Consolidated Identity, Disturbed Identity, and Lack of Identity. Consolidated identity stands for the feeling of oneself as a continuous, whole, and integrated entity over time and situations. Disturbed identity documents feelings of incoherence or uncertainty about one’s own identity, or a doubting of the authenticity of the social roles one takes up. Finally, Lack of Identity measures feeling inner emptiness, fragmentation, or feelings of non-existence. With the SCIM founded in the key developmental task typically for (late) adolescence, that is, developing a stable and coherent sense of self in interaction with the environment, it conceives of identity formation as an iterative task. As such, Disturbed Identity can be both pathological or part of a phase necessary for adaptive reorientation, while Lack of Identity represents the most unfavorable position (30).

### The current study: Aims and hypotheses

In the current study, we will investigate the utility of a combined ICD-11/DSM-5 AMPD view from a person-centered perspective. Our first aim is to investigate the potential advantages of the additional ANANKASTIA domain (not represented in the DSM-5 AMPD) as well as of the additional PSYCHOTICISM domain (not represented in the ICD-11 model) in the identification of meaningful pathological personality domain clusters based on the PID5BF + M. With regard to the ANANKASTIA domain specifically, we want to explore whether ANANKASTIA will consistently act in opposition to DISINHIBITION, as uni-dimensionally conceptualized in the DSM-5 AMPD, or that, in contrast, it will function independently of DISINHIBITION in the formation of meaningful pathological personality domain clusters. Based on existing research, we expect that the 2-cluster solution will generate (1) an overall-high and (2) an overall-low cluster. We expect that the 3-cluster solution will produce: (1) an Undercontrolled cluster, composed of high NEGATIVE AFFECTIVITY, high DISINHIBITION low ANANKASTIA, high ANTAGONISM, and high PSYCHOTICISM; (2) an Overcontrolled cluster, composed of high NEGATIVE AFFECTIVITY, high ANANKASTIA, low DISINHIBITION, low ANTAGONISM, and low PSYCHOTICISM; and (3) a Resilient cluster with overall-low scores. Given the presence of the additional ANANKASTIA domain, we will examine whether this will also give rise to a meaningful 4-cluster solution. For the 4- and 5-cluster solutions, our investigation will be explorative, except for the examination of a potential replication of the (4) the Confident-Disagreeable PID-5 cluster found by Bastiaens et al. (24), composed of low NEGATIVITY, high ANTAGONISM, high DISINHIBITION, and low PSYCHOTICISM.

Our second aim is to validate our 2- to 5-cluster solutions by investigating their relation with DSM-5 Section II personality disorder cluster A, B, and C scores. Based on existing literature (14), we expect that in the 2-cluster solution, the overall-high cluster will exhibit higher DSM-5 Section II cluster A, B, and C scores in comparison to the overall-low cluster. We expect that in the 3-cluster solution, the Undercontrolled cluster will show the highest DSM-5 Section II cluster A and B scores; that the Overcontrolled cluster will exhibit the highest DSM-5 Section II cluster C scores; and that the Resilient cluster will show the lowest DSM-5 Section II cluster A, B, and C scores.

Our third aim is to investigate the relation between pathological personality clusters stemming from the combined ICD-11/DSM-5 AMPD view, and Identity as a core feature of personality disorder severity in both the ICD-11 model and the DSM-5 AMD. Based on the present variable-centered and person-centered literature (9, 10, 24), we expect for the 2-cluster solution that the overall-high cluster will show lower Consolidated Identity and higher Disturbed Identity, and Lack of Identity scores in comparison to the overall-low cluster. For the 3-cluster solution, we expect: (1) the Resilient cluster to display the highest Consolidated Identity and the lowest Disturbed Identity and Lack of Identity scores; (2) the Undercontrolled cluster to display the lowest Consolidated Identity and the highest Disturbed Identity and Lack of Identity scores; and (3) the Overcontrolled cluster to be situated in-between. For the 4- and 5-cluster solutions, if indeed the PID-5 Confident-Disagreeable cluster by Bastiaens et al. (24) can be replicated, we expect it to display relatively favorable Consolidated Identity, Disturbed Identity, and Lack of Identity scores, as in the latter study it was associated with favorable SIPP-118 Stable Self Image scores [in contrast to unfavorable Trustworthiness and Responsible Industry scores (24)].

## Materials and methods

### Participants and procedure

A Flemish community sample of 242 subjects voluntarily participated in the current study. Participants were recruited by Master students in Psychology trough closed envelops stipulating age and gender according to the National Institute for Statistics, in order to obtain a population-representative sample. Of the total sample, 188 filled in the PID5BF + M, the Assessment of the DSM-IV personality disorders (ADP-IV) (32), and the Self-Concept and Identity Measure (SCIM) [(29), Dutch translation by Bogaerts et al. (30)]. Of the 188 participant sample, 95 (50%) identified themselves as female and 94 (50%) as male. Three (1.6%) participants attained no educational degree, nine (5%) only finished elementary school as the highest educational level obtained, 69 (36%) completed high school, and 57% successfully finished higher educational studies. Age ranged from 18 to 67 years, with a mean age of 43.59 (*SD* = 14.47). Participants were provided with written information explaining the aims of the current study, the guaranteed anonymity in participation, and signed an informed consent. The study was approved by the ethical committee of KU Leuven (SMEC).

### Measures

To assess the pathological personality domains combining the DSM-5 AMPD and ICD-11 personality model architecture, we used the Modified Personality Inventory for DSM-5—Brief Form Plus (PID5BF + M) (8) is a 36-item self-report questionnaire using a 4-point Likert-type scale (ranging from 0: *not at all true*, to 3: *entirely true*) that measures six pathological trait domains, each comprised of six items. The six pathological trait domains are: Negative Affectivity (NA), Detachment (D), Antagonism (A), Disinhibition (DIS), Anankastia (ANAN), and Psychoticism (P). The PID5F + M has been validated in 15 countries (4). Cronbach alpha coefficients in current study equaled 0.79 for NA, 0.79 for D, 0.81 for A, 0.79 for DIS, 0.83 for ANAN, and 0.80 for P.

To assess DSM-5, Section II PD clusters, we used the Assessment of the DSM-IV personality disorders (ADP-IV) (32) is a 94-item self-report questionnaire measuring the diagnostic criteria for the DSM-IV personality disorders in a dimensional way, using a 7-point Likert-type scale (1: *totally disagree*, to 7: *totally agree*). Its reliability and validity has been documented extensively (32, 33). Cronbach’s alpha coefficients in the current study equaled 0.89 for Cluster A, 0.93 for Cluster B, and 0.92 for Cluster C personality disorders.

To assess identity functioning, we used the Self-Concept and Identity Measure (SCIM) [(29), Dutch translation by Bogaerts et al. (30)] is a 27-item, self-report questionnaire that assesses Consolidated Identity (10 items), Disturbed Identity (11 items), and Lack of Identity (6 items) on a 7-point Likert-type scale (1: *completely disagree*; 7: *completely agree*). Its reliability and validity has been documented extensively (29, 30, 34). Cronbach’s alpha coefficients amounted to 0.64 for Consolidated Identity, 0.79 for Disturbed Identity, and 0.87 for Lack of Identity.

### Analyses

All analyses were performed by means of SPSS version 27. To explore the different cluster solutions, cluster analysis (35) was applied on the PID5BF + M domain scores using a two-step procedure, with a hierarchical cluster analysis using Ward’s method based on squared Euclidian distances in the first step, and an iterative k-means clustering procedure using the initial cluster centers as non-random starting points in the second step. Potential outliers were defined using a value of *Z* > 2.5 on any PID5BF + M domain. *R*^2^ was used as a measure of variance explained in each PID5BF + M domain by the respective cluster solution. PID5BF + M, ADP-IV, and SCIM Mean level differences between the clusters were investigated through Multivariate Analyses of Variance (MANOVAs). Pairwise comparisons between clusters were conducted by means of Scheffé’s *post-hoc* comparisons (*p* < 0.05).

## Results

### Cluster analyses and differences between clusters in PID5BF + M domain mean level Z-scores for each cluster solution

**FIGURE 1:**
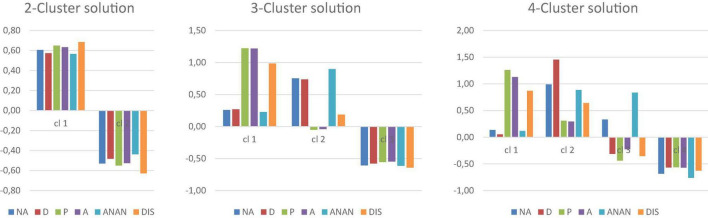
PID5BF + M two-, three-, and four-cluster solutions.

The respective 2-, 3-, and 4-cluster solutions are depicted in Figure 1. The MANOVA for the 2-cluster solution showed an overall effect of cluster membership on PID5BF + M domain scores [*Wilk’s Lambda* = 0.30, *F*_(6, 181)_ = 69.78, *p* < 0.001, *partial*η^2^ = 0.70], with the univariate ANOVA’s (Table 2) displaying significant differences between the clusters on each individual PID5BF + M domain, giving rise an (1) overall high (*n* = 86, 45.74%) and an (2) overall low (*n* = 102, 54.26%) cluster as expected. In the 2-cluster solution, *R*^2^ equaled 0.33 for NEGATIVE AFFECTIVITY, 0.28 for DETACHMENT, 0.36 for PSYCHOTICISM, 0.34 for ANTAGONISM, 0.25 for ANANKASTIA, and 0.45 for DISINHIBITION.

For the 3-cluster solution, an overall effect of cluster membership on PID5BF + M domain scores was found as well [*Wilk’s Lambda* = 0.13, *F*_(12, 360)_ = 52.03, *p* < 0.001, *partial*η^2^ = 0.63], again with a main effect of cluster membership on each individual PID5BF + M domain (Table 2). The first cluster showed high ANTAGONISM, high DISINHIBITION without elevation of ANANKASTIA, and high PSYCHOTICISM, but without elevation of NEGATIVE AFFECTIVITY, and was labeled the (1) Undercontrolled-but-not-fearful cluster (*n* = 43, 22.87%). The second cluster was characterized by high NEGATIVE AFFECTIVITY and high ANANKASTIA, without elevation of DISINHIBITION, ANTAGONISM, or PSYCHOTICISM, and by high DETACHMENT, and was labeled the (2) Overcontrolled cluster (*n* = 55, 29.55%). The third cluster displayed overall-low scores and was labeled the (3) Resilient cluster (*n* = 90, 47.87%). *Post-hoc* comparisons showed that: the (3) Resilient cluster scored significantly lower on all domains in comparison to the two other clusters; the (2) Overcontrolled cluster scored significantly higher on NEGATIVE AFFECTIVITY, DETACHMENT, and ANANKASTIA in comparison to both other clusters; the (1) Undercontrolled-but-not-fearful cluster scored significantly higher on ANTAGONISM, DISINHIBITION, and PSYCHOTICISM compared to both other clusters. In the 3-cluster solution, *R*^2^ equaled 0.37 for NEGATIVE AFFECTIVITY, 0.34 for DETACHMENT, 0.49 for PSYCHOTICISM, 0.49 for ANTAGONISM, 0.43 for ANANKASTIA, and 0.45 for DISINHIBITION.

For the 4-cluster solution, the MANOVA again displayed an overall effect of cluster membership on PID5BF + M domain scores [*Wilk’s Lambda* = 0.07, *F*_(18, 506.77)_ = 42.71, *p* < 0.001, *partial*η^2^ = 0.58], again with a main effect of cluster membership on each individual PID5BF + M domain (Table 2). The three former clusters were retained [(1) Undercontrolled-but-not-fearful: *n* = 38, 20.21%; (3) Overcontrolled: *n* = 33, 17.55%; (4) Resilient: *n* = 80, 42.55%], with the (3) Overcontrolled cluster now characterized by even more pronounced ANANKASTIA and NEGATIVE AFFECTIVY vs. low DISINHIBITION, and low DETACHMENT as well. The fourth cluster emerged as showing very high NEGATIVE AFFECTIVITY, very high DETACHMENT, high ANANKASTIA but also high DISINHIBITION, with moderate PSYCHOTICISM and moderate ANTAGONISM scores, and was labeled the (2) fearfully-detached, oscillating-between-control-and-disinhibition cluster (*n* = 37, 19.68%). *Post-hoc* comparisons showed that: the (4) Resilient cluster scored significantly lower on NEGATIVE AFFECTIVITY and ANANKASTIA in comparison to the other three clusters; the (3) Overcontrolled cluster did not statistically differ from the (4) Resilient cluster on DETACHMENT, ANTAGONISM, PSYCHOTICISM, and DISINHIBITION mean scores; the (2) fearfully-detached, oscillating-between-control-and-disinhibition cluster scored significantly higher on NEGATIVE AFFECTIVITY and DETACHMENT in comparison to the other three groups, did not statistically differ from the (3) Overcontrolled group in ANANKASTIA, but in contrast to the latter, showed a significantly higher DISINHIBITION score, situated in the same range as the DISINHIBITION score of the (1) Undercontrolled-but-not-fearful cluster. Finally, the (1) Undercontrolled-but-not-fearful group demonstrated significantly higher ANTAGONISM and higher PSYCHOTICISM than all other groups, and lower ANANKASTIA mean scores compared to the (2) fearfully-detached, oscillating-between-control-and-disinhibition cluster. The 4-cluster solution provided the most variance explained in each PID5BF + M domain, with *R*^2^ equaling 0.43 for NEGATIVE AFFECTIVITY, 0.58 for DETACHMENT, 0.51 for PSYCHOTICISM, 0.43 for ANTAGONISM, 0.53 for ANANKASTIA, and 0.44 for DISINHIBITION.

**TABLE 2:** PID5BF + M cluster solutions with PID5BF + M, ADP-IV, and SCIM means and standard deviations.

										*F*	*Partial* η^2^	*Post-hoc* comparisons *p* < 0.05

2-cluster solution												
		Cluster 1		Cluster 2								
		*M*	*SD*	*M*	*SD*							
**PID5BF + M**												
	*Z*(NA)	0.61	0.91	–0.53	0.72					90.59***	0.33	
	*Z*(D)	0.57	1.05	–0.48	0.62					73.31***	0.28	
	*Z*(P)	0.65	1.11	–0.55	0.39					103.45***	0.36	
	*Z*(A)	0.63	1.04	–0.53	0.56					94.82***	0.34	
	*Z*(ANAN)	0.57	0.89	–0.44	0.86					62.04***	0.25	
	*Z*(DIS)	0.68	0.88	–0.63	0.59					149.22***	0.45	
**ADP-IV**										
	Cl A	2.30	0.81	1.55	0.39					69.48***	0.27	
	Cl B	2.14	0.72	1.37	0.31					94.13***	0.34	
	Cl C	2.70	0.98	1.78	0.62					59.21***	0.24	
**SCIM**												
	CONS	5.27	0.55	5.73	0.68					24.87***	0.12	
	DIST	2.60	0.77	1.96	0.66					37.08***	0.17	
	LACK	2.15	0.90	1.49	0.68					31.93***	0.15	

**3-cluster solution**												
		**Cluster 1**		**Cluster 2**		**Cluster 3**						
		***M***	***SD***	***M***	***SD***	***M***	***SD***					

**PID5BF + M**												
	*Z*(NA)	0.26	0.79	0.75	0.94	–0.61	0.69			53.48***	0.37	2 > 1 > 3
	*Z*(D)	0.27	0.95	0.74	1.08	–0.58	0.47			48.41***	0.34	2 > 1 > 3
	*Z*(P)	1.22	1.16	–0.05	0.69	–0.56	0.39			13.96***	0.49	1 > 2 > 3
	*Z*(A)	1.22	0.91	–0.04	0.77	–0.55	0.56			14.79***	0.49	1 > 2 > 3
	*Z*(ANAN)	0.23	0.86	0.90	0.78	–0.61	0.70			8.26***	0.43	2 > 1 > 3
	*Z*(DIS)	0.98	1.00	0.19	0.71	–0.64	0.59			9.87***	0.45	1 > 2 > 3
**ADP-IV**										
	Cl A	2.38	0.90	2.16	0.67	1.50	0.37			36.73***	0.29	1, 2 > 3
	Cl B	2.41	0.82	1.82	0.44	1.34	0.29			67.94***	0.43	1 > 2 > 3
	Cl C	2.55	0.99	2.74	0.93	1.70	0.56			34.73***	0.27	1, 2 > 3
**SCIM**												
	CONS	5.23	0.52	5.33	0.59	5.76	0.67			13.75***	0.13	3 > 2, 1
	DIST	2.74	0.90	2.48	0.55	1.88	0.65			26.37***	0.23	1, 2 > 3
	LACK	2.11	1.04	2.10	0.72	1.46	0.70			14.99***	0.14	1, 2 > 3

**4-cluster solution**												
		**Cluster 1**		**Cluster 2**		**Cluster 3**		**Cluster 4**				
		***M***	***SD***	***M***	***SD***	***M***	***SD***	***M***	***SD***			

**PID5BF + M**												
	*Z*(NA)	0.14	0.73	0.99	0.95	0.33	0.79	–0.69	0.65	45.41***	0.43	2 > 3, 1 > 4
	*Z*(D)	0.05	0.73	1.45	0.80	–0.31	0.63	–0.57	0.52	85.84***	0.58	2 > 1, 3, 4; 1 > 4
	*Z*(P)	1.26	1.22	0.31	0.71	–0.44	0.50	–0.56	0.38	63.59***	0.51	1 > 2 > 3, 4
	*Z*(A)	1.12	0.86	0.30	1.00	–0.23	0.79	–0.57	0.53	45.92***	0.43	1 > 2 > 3, 4
	*Z*(ANAN)	0.12	0.84	0.89	0.83	0.84	0.60	–0.76	0.58	68.18***	0.53	2, 3 > 1 > 4
	*Z*(DIS)	0.87	1.01	0.64	0.78	–0.36	0.59	–0.63	0.62	48.22***	0.44	1, 2 > 3, 4
**ADP-IV**												
	Cl A	2.25	0.81	2.53	0.80	1.75	0.41	1.49	0.39	31.52***	0.34	1, 2 > 3, 4
	Cl B	2.29	0.76	2.11	0.70	1.64	0.36	1.31	0.28	38.14***	0.39	1, 2 > 3 > 4
	Cl C	2.32	0.78	3.20	1.07	2.22	0.64	1.67	0.55	36.55***	0.38	2 > 1, 3 > 4
**SCIM**												
	CONS	5.30	0.52	5.18	0.60	5.53	0.45	5.77	0.72	9.42***	0.14	4 > 1, 2
	DIST	2.59	0.81	2.60	0.77	2.49	0.61	1.84	0.62	16.55***	0.22	1, 2, 3 > 4
	LACK	2.00	0.99	2.43	0.80	1.68	0.57	1.45	0.72	13.96***	0.19	2 > 3, 4; 1 > 4

NA, Negative Affectivity; D, Detachment; P, Psychoticism; A, Antagonism; ANAN, Anankastia; DIS, Disinhibition; CONS, Consolidated Identity; DIST, Disturbed Identity; LACK, Lack of Identity. ***p < 0.001.

A 5-cluster solution yielded only five subjects in the fifth cluster, and was not included in the manuscript.

### Differences between clusters in ADP-IV cluster A, B, and C, and individual personality disorder scores for each cluster solution

The MANOVA for the 2-cluster solution showed an overall effect of cluster membership on ADP-IV personality disorder Cluster A, B, and C scores [*Wilk’s Lambda* = 0.65, *F*_(3, 183)_ = 33.41, *p* < 0.001, *partial*η^2^ = 0.35], with the univariate ANOVA’s (Table 2) displaying significant differences between the clusters on each ADP-IV personality disorder cluster. *Post-hoc* comparisons showed that the (1) overall-high cluster showed significantly higher ADP-IV Cluster A, B, and C mean scores in comparison to the (2) overall-low cluster, as expected. In addition, an overall effect of cluster membership on the individual personality disorder scores was found as well [*Wilk’s Lambda* = 0.60, *F*_(12,174)_ = 9.79, *p* < 0.001, *partial*η^2^ = 0.40], with the univariate ANOVA’s showing significant differences for each individual personality disorder, and the (1) overall-high cluster consistently scoring significantly higher than the (2) overall-low cluster (Appendix Table A1).

In the 3-cluster solution, a main effect of cluster membership was found as well [*Wilk’s Lambda* = 0.44, *p* < 0.001, *F*_(6, 364)_ = 30.71, *partial*η^2^ = 0.34], again with the univariate ANOVA’s (Table 2) displaying significant differences between the clusters on each ADP-IV personality disorder cluster. *Post-hoc* comparisons are shown in Table 1. The (3) Resilient cluster showed significantly lower mean scores on all ADP-IV Cluster scores in comparison to both other clusters; the (2) Overcontrolled cluster did not statistically differ from the (1) Undercontrolled-but-not-fearful cluster with regard to ADP-IV cluster A or cluster C mean scores; finally, the (1) Undercontrolled-but-not-fearful cluster showed significantly higher cluster B mean scores than the (2) Overcontrolled cluster, which in turn did so compared to the (3) Resilient cluster. In addition, an overall effect of cluster membership on the individual personality disorder scores was found as well [*Wilk’s Lambda* = 0.34, *F*_(24,346)_ = 10.47, *p* < 0.001, *partial*η^2^ = 0.42], again with the univariate ANOVA’s showing significant differences for each individual personality disorder. *Post-hoc* comparisons are listed in Appendix Table A1. Results paralleled the findings on the cluster level, with two exceptions. First, (1) Undercontrolled-but-not-fearful cluster did not significantly differ from the (2) Overcontrolled cluster with regard to the borderline personality disorder, in contrast to the other individual cluster B scores. Second, the (1) Undercontrolled-but-not-fearful cluster did significantly differ from the (2) Overcontrolled cluster with regard to the schizotypal personality disorder, in contrast to the other individual cluster A scores.

For the 4-cluster solution, the MANOVA again showed a main effect of cluster membership [*Wilk’s Lambda* = 0.42, *p* < 0.001, *F*_(9, 440.66)_ = 21.19, *partial*η^2^ = 0.25], again with the univariate ANOVA’s (Table 2) displaying significant differences between the clusters on each ADP-IV personality disorder clusters. *Post hoc* comparisons are shown in Table 1. The (4) Resilient cluster demonstrated, together with the (3) Overcontrolled cluster, significantly lower ADP-IV Cluster A mean scores in comparison to the other two clusters; the (4) Resilient cluster also showed significantly lower ADP-IV Cluster B and Cluster C mean scores compared to all other clusters; the (3) Overcontrolled cluster demonstrated a significantly lower ADP-IV Cluster B mean score than the (2) fearfully-detached, oscillating-between-control-and-disinhibition cluster, and the (1) Undercontrolled-but-not-fearful cluster, and a significantly lower ADP-IV cluster C score in comparison to the former; the (2) fearfully-detached, oscillating-between-control-and-disinhibition cluster did not statistically differ from the (1) Undercontrolled-but-not-fearful cluster on ADP-IV Cluster A of Cluster B mean scores, but did demonstrate a higher ADP-IV Cluster C mean score in comparison to the latter. For the individual personality disorder scores, an overall effect of cluster membership was found as well [*Wilk’s Lambda* = 0.31, *F*_(36,508.92)_ = 6.86, *p* < 0.001, *partial*η^2^ = 0.32], again with the univariate ANOVA’s showing significant differences for each individual personality disorder, and the *post-hoc* comparisons listed in Appendix Table A1. Results generally paralleled the findings on the cluster level.

### Differences between clusters in self-concept and identity measure scores for each cluster solution

For the 2-cluster solution, a main effect of cluster membership was found [*Wilk’s Lambda* = 0.77, *F*_(3, 179)_ = 17.61, *p* < 0.001, *partial*η^2^ = 0.23], with the (1) overall-high cluster showed significantly lower Consolidated Identity scores and significantly higher Disturbed, and Lack of Identity scores in comparison to the (2) overall-low cluster, as expected.

For the 3-cluster solution, a general effect of cluster membership was found as well [*Wilk’s Lambda* = 0.73, *F*_(6, 356)_ = 10.32, *p* < 0.001, *partial*η^2^ = 0.15], with the univariate ANOVA’s (Table 2) displaying significant differences between the clusters on all three SCIM subscale scores. *Post-hoc* comparisons showed the (3) Resilient cluster displaying significantly more Consolidated Identity and significantly less Disturbed Identity and Lack of Identity in the *post-hoc* comparisons.

For the 4-cluster solution, a general effect of cluster membership was again found [*Wilk’s Lambda* = 0.69, *F*_(9, 430.92)_ = 8.02, *p* < 0.001, *partial*η^2^ = 0.12], again with significant differences between the clusters on all three SCIM scores (Table 2). *Post-hoc* comparisons showed that: the (4) Resilient cluster displayed more Consolidated Identity in comparison to the (1) Undercontrolled-but-not-fearful cluster, and in comparison to the (2) fearfully-detached, oscillating-between-control-and-disinhibition cluster, with the Overcontrolled cluster (3) taking up an intermediate position. The (4) Resilient cluster showed significantly less Disturbed Identity in comparison to all other clusters, that did not significantly differ from each other. Finally, the (4) Resilient cluster together with the (3) Overcontrolled cluster showed significantly less Lack of Identity than the (2) fearfully-detached, oscillating-between-control-and-disinhibition cluster, with the (1) Undercontrolled-but-not-fearful cluster in between.

## Discussion

In the current study, we investigated the utility a combined ICD-11/DSM-5 AMPD view from a person-centered perspective, including an additional ANANKASTIA domain (not represented in the DSM-5 AMPD) as well as a PSYCHOTICISM domain (not represented in the ICD-11 model) in the identification of meaningful pathological personality domain clusters. We then validated these clusters by investigating differences between them in mean DSM-5 Section II cluster A, B, and C personality disorder scores, and finally investigated whether cluster membership was able to differentiate between levels of identity functioning, a key feature of personality disorder severity in both the ICD-11 model and the DSM-5 AMD.

Regarding the first goal, the current study demonstrates meaningful cluster solutions at the 2-, 3-, and 4-cluster solution level in line with expectations, with the 4-cluster solution explaining the most variance in the PID5BF + M clustering variables. Specifically, the use of the PID5BF + M with its separate ANANKASTIA domain allows for the identification of a classical Overcontrolled cluster which the DSM-5 AMPD PID-5 has not been able to detect (24, 36). In addition, in the 4-cluster solution, the separate ANANKASTIA domain allowed to identify a personality domain cluster exhibiting high DISINHIBITION and high ANANKASTIA simultaneously, which is not possible in a classical DSM-5 AMPD PID-5 view, as DISINHIBITION and ANANANKASTIA are considered opposite extremes on one dimension. As such, our results contribute to the debate on the DSM-5 AMPD/ICD-11 personality pathology architecture from a cluster analysis perspective, advocating the validity of a separate ANANKASTIA domain apart from DISINHIBITION (37). Surprisingly, our Undercontrolled type in the 3-cluster solution and preserved in the 4-cluster solution, did not show heightened NEGATIVE AFFECTIVITY. In effect, it thereby resembled the Confident-Disagreeable cluster found in Bastiaens et al. (24). Where the use a non-clinical sample may indeed allow an Antagonistic-Disinhibited, but not fear-ridden personality type to surface, Bastiaens et al. (24), using a non-clinical sample as well, did find a more typical (i.e., high NEGATIVE AFFECTIVITY) Undercontrolled cluster next to their Confident-Disagreeable cluster. However, both personality types were part of a 6-cluster solution using a different technique, whereas in the current study the specific constellation of the personality types at this level could not be investigated as one cluster in the five-cluster solution did not provide enough subjects to continue.

Regarding the second goal, the current study shows that DSM-5 Section II cluster A, B, and C personality disorder scores differed according to PID5BF + M cluster membership in the expected way. These findings are consistent with Bohane et al. (14) general descriptions of the personality clusters, and are helpful as a potential crosswalk from the classical DSM-IV/DSM-5 Section II model to a person-centered take on the combined DSM-5 AMPD/ICD-11 model. While in the 3-cluster solution, the Undercontrolled-but-not-fearful cluster showed a significant higher DSM-5 Section II cluster B mean score as expected, the additional fearfully-detached, oscillating-between-control-and-disinhibition cluster in the 4-cluster solution scored equally high on DSM-5 Cluster A and even significantly higher on both DSM-5 Section II Cluster B and Cluster C compared to the Undercontrolled-but-not-fearful cluster. This paralleled findings with regard to the Identity measures described below. Of note, on the level of the individual personality disorder scores in the 3-cluster solution, the finding that that de Undercontrolled-but-not-fearful cluster did not differ from the Overcontrolled cluster in its borderline personality disorder score, is consistent with the unique position the borderline personality disorder occupies in the HiTOP-model, i.e., loading on both the Internalizing as well as on the Antagonistic-Externalizing spectrum, while the other three cluster B personality disorders only load on the latter (38, 39).

Finally, our current study demonstrated that clinical-developmental Identity levels, a core feature of DSM-5 AMPD and ICD-11 personality disorder severity, differed according to PID5BF + M cluster membership. At the same time, we found that in the 4-cluster solution, the fearfully-detached, oscillating-between-control-and-disinhibition cluster equaled the high scores of the Undercontrolled-but-not-fearful cluster for all three SCIM-scores. These findings correspond to Bastiaens et al. (24) reporting of on their PID-5 Anxiously-Detached cluster displaying equal or even worse SIPP-118 scores in comparison to their Undercontrolled (including high NEGATIVE AFFECTIVITY) PID-5 cluster.

Of specific interest, the Overcontrolled cluster manifested similar Disturbed Identity mean scores as the Undercontrolled-but-not-fearful and the fearfully-detached, oscillating-between-control-and-disinhibition cluster, while contrasting itself from both by its more favorable Lack of Identity mean score, in fact aligning with the Resilient cluster. Given the theoretical background of the SCIM, these findings provide validation for PID5BF + M personality type differentiation with regard to clinical-developmental identity functioning. Second, our Undercontrolled-but-not-fearful cluster showed among the highest SCIM Disturbed as well as Lack of Identity scores, in contrast to Bastiaens et al. (24) Confident-Disagreeable Cluster (displaying relatively favorable SIPP-118 Stable Self Image scores). A major difference in comprising domains is that our Undercontrolled-but-not-fearful cluster, while equally presenting with low NEGATIVE AFFECTIVITY, high ANTAGONISM, and high DISINHIBITION, also displayed high PSYCHOTICISM, which Bastiaens et al.’s Confident-Disagreeable Cluster did not. As such, current findings also contribute to the clinical relevance of the P-domain, in line with Benzi et al. (40) from a variable-centered perspective. With the SCIM representing a clinical-developmental approach, current differentiating, cross-sectional findings are encouraging for future research in which the role of the identity formation *process* can be investigated as a candidate-mediating factor in the well-documented correlations between personality clusters on the one hand, and life outcomes on the other (41).

Besides the strengths of our study, some limitations need to be addressed. As a first limitation, the current research was conducted in a non-clinical sample, so future studies are needed to see if the current cluster-solutions can be replicated in clinical samples. Specifically, whether this would allow for the emergence of an Undercontrolled cluster that shows high Negative Affectivity in its configuration. Moreover, our non-clinical sample was limited in size and balanced for gender and age, but not for educational level, which turned out relatively high (with 57% having successfully finished higher educational studies in comparison to the population-representative 52.4%)^1^. Future investigations using larger samples are needed to find out if our results at the 2-, 3-, and 4-cluster solution level can be replicated, and whether a fifth cluster would emerge if the number of participants in the sample would be substantially increased. As a second limitation, we cross-sectionally investigated the effect of PID5BF + M cluster membership on a clinical-developmental conceptualization of Identity. While future studies obviously also need to focus on the Interpersonal part of the Personality Disorder Severity dimension, it would be most interesting to investigate differences in Identity formation between PID5BF + M clusters using longitudinal designs.

Notwithstanding the above, to our knowledge the current study is the first to investigate personality clusters based on the combination of the DSM-5 AMD/ICD-11 personality model, and the first to differentiate these clusters with regard to a clinical-developmental operationalization of Identity (problems), using 2-, 3-, and 4-cluster solutions. Future longitudinal research can focus on the role of the identity formation *process* as a potential mediating or moderating factor in the correlations between personality types on the one hand, and life outcomes on the other.

## Data availability statement

The raw data supporting the conclusions of this article will be made available by the authors, without undue reservation.

## Ethics statement

The studies involving human participants were reviewed and approved by Ethical Committee of KU Leuven (SMEC). The patients/participants provided their written informed consent to participate in this study.

## Author contributions

TB: writing—original draft and writing—review and editing. AB: data collection. KL: review and editing. DS: data-analyses. LC: data-analyses and review and editing. All authors contributed to the article and approved the submitted version.

## Footnotes

1
https://statbel.fgov.be/en/themes/work-training/training-and-education/level-education

## Appendix

**TABLE A1:** Means and standard deviations of individual ADP-IV personality disorder scores for each PID5BF + M cluster solution.

										*F*	*Partial* η^2^	*Post-hoc* comparisons *p* < 0.05

2-cluster solution												
		Cluster 1		Cluster 2								
		***M***	***SD***	***M***	***SD***							
**ADP-IV**												
	PAR	2.38	1.08	1.55	0.56					45.71***	0.20	
	SZ	2.35	1.00	1.69	0.58					31.46***	0.15	
	ST	2.18	0.84	1.39	0.39					70.57***	0.28	
	AS	1.68	0.81	1.17	0.27					35.44***	0.16	
	BDL	2.37	0.88	1.58	0.52					55.93***	0.23	
	HIS	2.34	0.99	1.37	0.38					81.88***	0.31	
	NAR	2.17	0.81	1.37	0.40					76.62***	0.29	
	AV	2.65	1.31	1.70	0.79					37.42***	0.17	
	DEP	2.35	0.90	1.57	0.60					49.96***	0.21	
	OC	3.09	1.08	2.08	0.75					56.39***	0.23	

**3-cluster solution**												
		**Cluster 1**		**Cluster 2**		**Cluster 3**							
		***M***	***SD***	***M***	***SD***	***M***	***SD***					

**ADP-IV**												
	PAR	2.56	1.22	2.15	0.85	1.50	0.52			26.84***	0.23	1, 2 > 3
	SZ	2.23	0.97	2.36	0.99	1.66	0.54			15.32***	0.14	1, 2 > 3
	ST	2.35	0.91	1.96	0.68	1.34	0.35			42.74***	0.32	1 > 2 > 3
	AS	2.05	0.97	1.30	0.31	1.16	0.27			43.37***	0.32	1 > 2, 3
	BDL	2.40	0.97	2.27	0.75	1.52	0.50			30.85***	0.25	1, 2 > 3
	HIS	2.70	1.10	1.92	0.66	1.32	0.35			60.21***	0.40	1 > 2 > 3
	NAR	2.50	0.88	1.78	0.55	1.35	0.40			55.75***	0.38	1 > 2 > 3
	AV	2.45	1.23	2.74	1.31	1.61	0.72			22.17***	0.19	1, 2 > 3
	DEP	2.35	0.97	2.25	0.82	1.52	0.58			24.56***	0.21	1, 2 > 3
	OC	2.87	1.06	3.23	1.01	1.96	0.67			39.31***	0.30	2, 1 > 3

**4-cluster solution**												
		**Cluster 1**		**Cluster 2**		**Cluster 3**		**Cluster 4**					
		***M***	***SD***	***M***	***SD***	***M***	***SD***	***M***	***SD***			

**ADP-IV**												
	PAR	2.42	1.12	2.49	1.10	1.87	0.65	1.47	0.52	18.55***	0.23	2 > 3, 4; 1 > 4
	SZ	2.09	0.91	2.79	0.97	1.77	0.65	1.67	0.58	19.68***	0.24	2 > 1, 3, 4; 1 > 4
	ST	2.24	0.85	2.30	0.81	1.61	0.51	1.33	0.35	31.07***	0.34	2, 1 > 3, 4
	AS	1.99	0.88	1.45	0.71	1.28	0.35	1.16	0.28	19.52***	0.24	1 > 2, 3, 4
	BDL	2.27	0.87	2.60	0.93	1.91	0.52	1.50	0.49	25.07***	0.29	2 > 3 > 4; 1 > 4
	HIS	2.51	1.07	2.32	0.96	1.71	0.48	1.29	0.33	33.70***	0.36	1, 2 > 3, 4; 3 > 4
	NAR	2.38	0.86	2.07	0.79	1.66	0.52	1.32	0.35	30.70***	0.34	1 > 3, 4; 2 > 4
	AV	2.19	1.05	3.32	1.41	2.01	0.84	1.60	0.72	26.19***	0.30	2 > 1, 3, 4; 1 > 4
	DEP	2.13	0.85	2.67	0.94	1.88	0.74	1.50	0.51	23.20***	0.28	2 > 1, 3, 4; 1 > 4
	OC	2.64	0.84	3.61	1.15	2.78	0.76	1.91	0.65	37.02***	0.38	2 > 3, 1 > 4

PAR, paranoid personality disorder; SZ, schizoid personality disorder; ST, schizotypal personality disorder; AS, antisocial personality disorder; BDL, borderline personality disorder; HIS, histrionic personality disorder; NAR, narcissistic personality disorder; AV, avoidant personality disorder; DEP, dependent personality disorder; OC, obsessive-compulsive personality disorder. ****p* < 0.001.
